# Genetic characterisation of *Cryptosporidium parvum* in dairy cattle and calves during the early stages of a calving season

**DOI:** 10.1016/j.crpvbd.2023.100160

**Published:** 2023-12-01

**Authors:** Paul M. Bartley, Johan H. Standar, Frank Katzer

**Affiliations:** Moredun Research Institute, Pentlands Science Park, Bush Loan, Edinburgh, EH26 0PZ, United Kingdom

**Keywords:** *Cryptosporidium parvum*, Dairy, Cattle, Calves, Neonatal

## Abstract

*Cryptosporidium parvum* is a causative agent of cryptosporidiosis, an infectious gastroenteritis in neonatal ruminants, which can be fatal in severe cases. The aim of this study was to determine the prevalence of infections in dairy cattle/calves during the early stages of a calving season and the species/genotypes of the *Cryptosporidium* present. Faecal samples collected from pre- and post-partum dams (*n* = 224) as well as calves from age ∼1 day onwards (*n* = 312) were examined. Oocysts were concentrated, DNA extracted and tested by *Cryptosporidium* 18S rRNA gene PCR and sequencing, while genotypes of *C. parvum* were determined by *gp60* and VNTR analysis. Results showed that 31.3% and 30.4% of pre- and post-partum dams tested positive for *Cryptosporidium*, respectively. In the adults, *C. parvum* (*n* = 52), *C. bovis* (*n* = 4) and *C. andersoni* (*n* = 19) were identified, while in the calves 248 out of 312 (79.5%) were PCR-positive for *C. parvum*. The proportion of positive calf samples was significantly higher (*P* < 0.0001) than the proportion of positive adult cattle during the first seven weeks of the calving season. In adult cattle, three distinct *gp60* genotypes were identified, a predominant genotype IIaA15G2R1 (*n* = 36) and genotypes IIaA15R1 (*n* = 2) and IIaA14G2R1 (*n* = 1). In the calves, only genotype IIaA15G2R1 was detected (*n* = 125). Although *C. parvum* was observed in adult cattle two weeks after the start of the calving season, the predominant genotypes were not detected until Week 4 in both adults and calves, meaning it is still unclear whether adult cattle are the initial source of *C. parvum* infections on the farm. Historically calves on this dairy farm demonstrated the IIaA19G2R1 genotype, which, has now clearly been replaced with the IIaA15G2R1 genotype that is now found in both adults and calves. During the study season, significantly higher levels of neonatal calf mortality were observed compared to the seasons before (*P* = 0.046) and after (*P* = 0.0002). This study has shown comparable levels of *C. parvum* infection in both pre- and post-partum dams but higher levels of infection in neonatal calves.

## Highlights

• The farm had a predominant *C. parvum* MLVA-profile in calves and adult cattle faeces.
• No significant difference in *C. parvum* shedding in adult cattle pre- and post-partum.
• The predominant genotype appeared in adult cattle and calves at the same time.
• Once *C. parvum* was established, infections of new calves occurred almost directly.
• The predominant *C. parvum* genotype on the farm switched after at least 10 years.

## Graphical abstract

**Figure:**
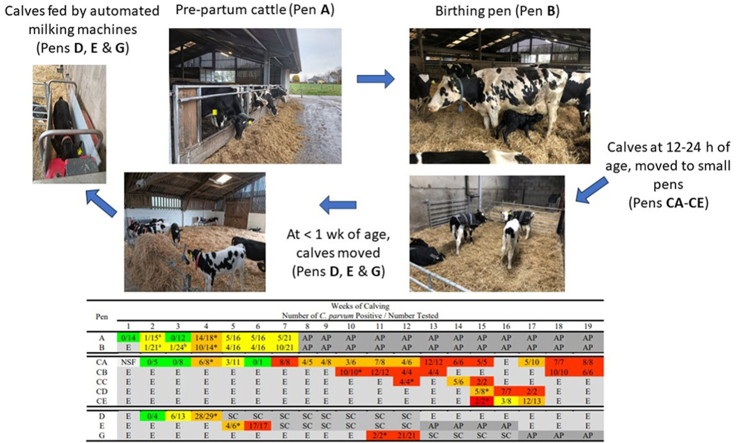

## 1 Introduction

Cryptosporidiosis is one of the most commonly diagnosed causes of infectious enteritis in neonatal dairy calves (Shaw et al., 2021). From a One Health perspective, the most important species is *Cryptosporidium parvum*, not only because it can cause potentially fatal disease in neonatal ruminants, but as it is also zoonotic and a major cause of human cryptosporidiosis worldwide, with young children being especially vulnerable (Helmy et al., 2015). There are three other main species of *Cryptosporidium* that are found in cattle, *C. bovis*, C*. ryanae* which are generally found in older calves (4 weeks to ≤ 6 months of age) (Thomson et al., 2019b), while *C. andersoni* is usually found in post-weaned calves and adult animals (Wang et al., 2011). Currently, there are at least 47 recognised species of *Cryptosporidium*, as well as many more genotypes infecting a range of host species, from mammals and birds to amphibians and reptiles (Prediger et al., 2021; Ryan et al., 2021; Huang et al., 2023; Tůmová et al., 2023).

*Cryptosporidium parvum* is the only species infecting cattle that is associated with clinical disease in naturally infected neonatal calves. The clinical signs of cryptosporidiosis in calves include profuse watery diarrhoea, dehydration, loss of appetite and depression, which in severe cases can result in calf mortality (Thomson et al., 2017). This susceptibility to *C. parvum* means that following infection young calves can shed huge numbers (3.89 × 10^10^) of oocysts in a very short period (6–12 days) (Nydam et al., 2001). Although older calves (≥ 6 months) and adult cattle are known to be naturally infected with *C. parvum* (Shaw et al., 2021) these infections are generally sub-clinical/asymptomatic, with infected adult animals shedding much lower numbers of oocysts, compared to neonates.

There is very little information available for cattle as to whether parturition influences levels of *C. parvum* oocyst shedding. In one study no oocysts were found in faecal samples collected from dams 12 h after calving (Atwill and Pereira, 2003), while another study found that 6/32 (18.8%) periparturient cows (within ± 7 days of giving birth) were excreting *C. parvum* oocysts (Castro-Hermida et al., 2002). In another study, Thomson et al. (2019a) found that four weeks before calving 27.3% (*n* = 57) of adult cattle samples tested positive for *Cryptosporidium*. These data suggest that levels of oocyst shedding in/around pregnancy in cattle are relatively low.

We were interested in investigating what role (if any) the dams play in the initiation and spread of *C. parvum* infections during the early stages of a calving season on a commercial working dairy farm. We were also interested in the species and genotypes of *Cryptosporidium* present in both adults and calves, as well as the timings of infections. To do this we collected faecal samples from adult cattle in the last two weeks of pregnancy and from post-partum cattle. We also collected samples from the calves once they had been separated from the dams (∼1 day of age).

## 2 Materials and methods

### 2.1 Study design and farm setup

The study was carried out on a working dairy farm located in Midlothian (Central Scotland, UK). The farm contains a closed herd, with all replacement stock being bred on-site using artificial insemination of sexed semen from known genetic lines, while the cattle not being used to produce replacement calves were bred using beef cattle semen. The farm has a history of *C. parvum* infection in their cattle (Thomson et al., 2019b) and also in farm-based infections of people (Gait et al., 2008) The calving season takes place from mid-August to late March. No calves are born between April and mid-August. At the end of the calving season, all the pens are cleared, cleaned, disinfected, and then left clean and empty until the next calving season.

The cattle and calves were fully managed by the farm staff (stockmen), with their movement between pens being coordinated as per normal farm practices. During the study period, all the animals were housed in concrete sided pens that contained straw bedding, which was changed routinely during the calving season. The straw in all of the pens (adults and calves) was refreshed 3–4 times a week. Each batch calf pen was mucked out between batches of calves and pressure washed and disinfected with Downland Blitz (Downland Partners in Animal Health, Carlisle, UK). The adult cattle pens were mucked out on a monthly basis.

The adult cattle sampled during the present study were divided into two separate pens. All dams are vaccinated with Rotovec (MSD Animal Health, Milton Keynes, UK), 4–8 weeks prior to calving. Pen A contained pre-partum dams in the last 1–2 weeks of pregnancy. Once a dam showed signs of labour, they were moved into Pen B, where they were left until they gave birth. Calves were usually removed from Pen B within approximately 12 h of birth. Post-partum dams remained in Pen B for 2–5 days, to ensure that there were no post-partum complications, after which time the dams were returned to the herd.

Following removal from their mothers (Pen B), the calves were initially housed in small groups (1–7 calves) in Pens CA - CE for 1–7 days (aim of 2–3 days only) where the calves were bottle-fed by the stockmen, before being moved to large group pens of up to 25 calves per pen (Pens D, E and G), where the calves were housed for the next 10–11 weeks.

Pens CA, CB and CC are located directly adjacent to Pen B, while Pens CD and CE are in a separate building and had not been used to house calves for several years prior to this calving season. Pens D and E are opposite to each other in a separate building away from the newborn and adult cattle. Pen G was also situated in another separate building away from all the other animals.

Once in the larger group pens (Pens D, E and G) each calf was fitted with a calf-feeder transponder collar and fed using automated milk machines, which monitored and limited the amount of milk each animal received. This system also allowed the farm staff to identify calves that were not feeding sufficiently and intervene when needed. A schematic diagram of the farm is provided in Supplementary Fig. S1.

Data were also collected on the total number of live/dead calves born as well as examining levels of calf mortality (before the age of 42 days), during the entire calving season.

Clinical observations were made by the scientist between weeks 5–19 whilst collecting samples from the pens; these observations focused particularly on the neonatal calves and included information on the general demeanour of the animals in specific pens and when clear signs of diarrhoea were seen.

### 2.2 Sample collection

#### 2.2.1 Adult samples

Adult cattle faecal samples (*n* = 224) were collected directly from the floor of each pen (Pen A and Pen B). For each sample, approximately 100 g of faeces was collected directly into a plastic bag, which was sealed until processing, usually within 1–5 days. Samples were collected randomly from different locations around the entirety of each pen. Each sample was appropriately labelled and then stored at 4 °C until processing.

#### 2.2.2 Calf samples

Calf faecal samples were also collected directly from the floor of each pen. For each individual sample, as large a quantity of faeces as possible was collected and placed into a labelled plastic bag. Each calf pen was thoroughly searched (usually by two or more scientists) to try to collect as many faecal samples as possible at each sampling time point. In the laboratory, the faeces were weighed, mixed with an equal volume of water and then transferred into a plastic tube. Once diluted, the samples were thoroughly mixed and then stored at 4 °C for 24–48 h prior to processing, to allow the faecal material to soften, aiding oocyst recovery.

### 2.3 Concentration of oocysts

#### 2.3.1 Adult samples

The oocysts in adult faecal samples (∼50 g) were concentrated using the acid flocculation and saturated salt floatation method previously described by Wells et al. (2016) with the following alterations. The pellets from the acid flocculation were resuspended in a 50 ml tube containing 30 ml saturated salt and 7.5–10 ml of water was trickled on top of the salt solution prior to centrifugation. The layer containing the oocysts was removed with a disposable plastic pipette and made up to a total volume of 25 ml in water prior to centrifugation. The final dilution volume of T1 buffer (Macherey-Nagel tissue kit) was 200–400 μl depending on the size of the pellet. Samples were stored at this stage at −20 °C prior to DNA extraction.

#### 2.3.2 Calf samples

As the calf samples were generally much smaller in volume than the adult samples, acid flocculation was not used. On arrival in the laboratory, all the bedding material (i.e. straw) was removed from the calf sample, which was then weighed and diluted with an equal volume of water (as described above in Section 2.2.2). For samples where the final diluted faecal volume was less than 15 ml, the entire sample was used for salt floatation. For samples where ≥ 15 ml of diluted faeces was available, then 15 ml was taken forward to saturated salt floatation (as described in Section 2.3.1).

### 2.4 DNA extraction

Prior to DNA extraction, each sample in T1 buffer underwent 10 cycles of freezing/thawing in liquid nitrogen/56 °C water bath, to disrupt the oocysts walls. The samples were then processed using a modified Macherey-Nagel NucleoSpin tissue kit (Macherey-Nagel, Düren, Germany) protocol, according to the manufacturerʼs instructions with the following amendments: the samples were incubated with 25 μl (10 mg/ml) proteinase K overnight (∼16 h). The samples were then vigorously vortexed and centrifuged at 14,000× *g* for 1 min and 200 μl of the resultant supernatant was removed into a fresh microfuge tube prior to further processing. For the final elution, each DNA sample was eluted in 50 μl of DNase/RNase-free water (dH_2_O). All DNA samples were stored at −20 °C prior to PCR analysis. An extraction control (dH_2_0) was processed with every batch of 23 samples to ensure that no cross-contamination occurred.

### 2.5 PCR amplification of 18S rRNA gene for *Cryptosporidium* spp. and *C. parvum*

The 18S nested PCR protocol used in this study was previously described (Brook et al., 2009; Wells et al., 2016; Thomson et al., 2019a) with the following modifications. General *Cryptosporidium*-specific 18S primers (AL1687: 5′-TTC TAG AGC TAA TAC ATG CG-3′ and AL1691: 5′-CCC ATT TCT TCG AAA CAG GA-3′) were used for the primary amplification. Overall, the PCRs were designed to amplify a *Cryptosporidium* spp. 18S fragment (840 bp) and a *C. parvum* 18S fragment (305 bp).

Each reaction consisted of 20 μl, including 2 μl of a 10× PCR buffer (45 mM of Tris-HCl pH 8.8, 11 mM of (NH_4_)_2_SO_4_, 4.5 mM of MgCl_2_, 4.4 μM of EDTA pH 8.0, 113 μg/ml BSA, 1 mM of each dNTP (dATP, dCTP, dGTP, dTTP)), 0.15 μl of *Taq* DNA polymerase (5 U/μl) (GenScript, Piscataway, NJ, USA) and 1 μl of each appropriate forward and reverse primers (10 μM), 13.85 μl of dH_2_O and 2 μl of DNA.

In the secondary amplification, a multiplex PCR was run with the following primers (AL1598: 5′-GGA AGG GTT GTA TTT ATT AGA TAA AG-3′, AL3032: 5′-AAG GAG TAA GGA ACA ACC TCC A-3′ and CphF: 5′-AGA GTG CTT AAA GCA GGC ATA-3′) included in the reaction mixture and only 12.85 μl of dH_2_0 was added. Furthermore, the entire primary nPCR product was diluted with 100 μl of dH_2_0 and 2 μl of the diluted amplicon was used in the secondary amplification. All PCR reactions were performed in triplicate; a sample was considered positive if at least 1/3 replicates tested positive. A negative control (dH_2_0), a DNA extraction control, along with a positive control for *C. parvum* DNA were included with each batch of samples. All samples that tested positive with the *Cryptosporidium* spp. primers but negative with the *C. parvum* primers were sent for sequencing to confirm the species of the *Cryptosporidium* present. A proportion of the samples that gave positive results using the *C. parvum* primers were also sent for sequencing to confirm the specificity of the primers. Results from each plate of PCR were only accepted if all of the positive, negative and extraction controls produced the expected result.

### 2.6 *gp60* PCR

Samples that produced a positive result using the *C. parvum*-specific 18S primers were further analysed using a nested *gp60* PCR, which has been previously described by Brook et al. (2009) and amplifies a final fragment of 375 bp. The PCR amplifications were completed in 20 μl reactions using the same basic master mix as previously described (Section 2.5), the forward and reverse primers (GP60_1F: 5′-ATA GTC TCC GCT GTA TTC-3 and GP60_1R: 5′-GAG ATA TAT CTT GGT GCG-3′) for the primary amplification and the primers for the secondary amplification (GP60_2F: 5′-TCC GCT GTA TTC TCA GCC-3′ and GP60_2R: 5′-CGA ACC ACA TTA CAA ATG AAG-3′). The entire primary PCR amplicon was diluted with 100 μl of dH_2_0, before 2 μl was used for the secondary amplification. Each sample was tested in triplicate, along with negative, extraction and positive controls for *C. parvum.*

The cycling conditions for both the 18S and the *gp60* PCR amplifications were 3 min at 94 °C, followed by 35 cycles of 45 s at 94 °C, 45 s at 55 °C and 1 min at 72 °C and a final extension step of 7 min at 72 °C. The completed PCR amplifications were held at 10 °C.

### 2.7 Variable Number Tandem Repeat (VNTR) multiplex PCR

Samples that produced positive results from the *gp60* PCR, where a clear *gp60* genotype was identifiable (using sequence analysis, see Section 2.10), were further analysed using the Variable Number Tandem Repeat (VNTR) PCR. The details for the VNTR PCR have been previously published as part of a Multi Locus VNTR Analysis (MLVA) typing scheme for *Cryptosporidium parvum* (Robinson et al., 2022). In brief, the PCRs comprise seven sets of primers, that are divided into two separate multiplex PCRs (one containing four primer sets and the other with three primer sets; Table 1). Each primer set was labelled with a unique fluorescent dye (PET, VIC, NED or FAM) and generated fragments of different sizes (Table 1). Each PCR reaction had a total volume of 25 μl, containing 12.5 μl of the 2× Type-it Microsatellite PCR master mix (Qiagen, Hilden, Germany), 2.5 μl of a 10× primer mix, 8 μl of dH_2_O and 2 μl of DNA. The 10× primer mix was composed of each appropriate set of primers used in the 4-plex and 3-plex PCRs, all at a concentration of 2 μM. Each PCR reaction was performed in a single well, a negative (dH_2_0) and a positive control of *C*. *parvum* DNA were used with each batch of samples. The PCR cycling conditions were: 5 min at 95 °C, 30 cycles of 30 s at 95 °C, 90 s at 63 °C and 30 s at 72 °C, with a final extension of 30 min at 60 °C. The PCR was then held at 4 °C.

**Table 1:** VNTR primers.

Primer	Sequence (5′-3′)
**4-plex primers**	
cgd1_470_1429F (F)	**PET-**CTCAGGAAGAGGAAGATACGG
cgd1_470_1429R (R)	GGAAGGTATGGCAGCAAAAG
cgd4_2350_796F (F)	**VIC**-GGGTCAATCAGGCATGAGC
cgd4_2350_796R (R)	TTGCATGTTTATCATATTATTTCCCAT
cgd8_4440_NC_506F (F)	**NED-**CTCAATATTTTTTCCACACCTGAAC
cgd8_4440_NC_506R (R)	ACTGCCTGAGAAAGGAACCA
cgd8_4840_6355F (MM19) (F)	**FAM**-GTTCCAGGAATATTTGATTCTGC
cgd8_4840_6355R (MM19) (R)	CTCCTACGCCAACTCCTA
**3-plex primers**	
cgd5_10_310F (MSF) (F)	**VIC**-AAGGTGAAGGAATCAAAGGC
cgd5_10_310R (MSF) (R)	TTTGTCCTTCTTGCCCTCGG
cgd5_4490_2941F (F)	**FAM**-CAGTGAATAACTCTGAACGGAAC
cgd5_4490_2941R (R)	TTGATTTTGGGTTCGGTATTG
cgd6_4290_9811F (F)	**NED-**CATTGGAACGTAAACAAAACCA
cgd6_4290_9811R (R)	CTAGCCGAATCTGGCGGTAT

### 2.8 Agarose gel electrophoresis

All secondary 18S and *gp60* PCR products were analysed by agarose gel electrophoresis using 1.5% agarose (in 1× TAE buffer) containing Gel Red at 1:10,000 dilution (Biotium, Fremont, CA, USA) with a run time of 90 min at 120 V. The VNTR PCR products were analysed using a 2.5% gel and were run at 70 V for 3 h. For each sample, 8 μl of the amplicon was loaded onto the gel, along with a 100 bp-1.5 Kb DNA molecular weight marker XIV (Roche, Welwyn Garden City, UK) and was then photographed under UV light (GBox).

### 2.9 PCR purification and sequence analysis

Prior to sequencing, PCR amplicons were purified using the Promega Wizard SV gel and PCR clean-up system (Promega, Southampton, UK) according to the manufacturerʼs instructions. The concentration of DNA for each sample was determined using spectrophotometry (NanoDrop One, Thermo Fisher, Waltham, MA, USA). The purified amplicons were stored at 4 °C for the short term and at −20 °C for long term.

For sequencing, ∼100 ng of DNA and 2 μl of appropriate primer (10 mM) in a final volume of 17 μl were sent for Sanger sequencing (MWG Eurofins). A consensus was created for each sample using overlapping forward and reverse sequences (DNASTAR program SeqMan Pro version 17; DNASTAR, USA). *Cryptosporidium* species were confirmed using NCBI (National Centre for Biotechnology Information) BLAST, while the *gp60* genotypes were determined by manually counting the number of trinucleotide repeats present in specific conserved regions of sequence (Chalmers et al., 2019).

### 2.10 Fragment size analysis

The (VNTR) multiplex PCR products (∼10 μl) were sent to the University of Dundee (DNA Sequencing and Services, UK), for fragment size analysis. The fragment size data were analysed using Geneious Prime software, version 2022 (Biomatters, Auckland, New Zealand). Each allele was identified based on fluorescence, amplicon size and the number of repeat units, which were used to generate the multilocus genotype (MLG) of the sample. The MLG was found for each of the seven fragments, with the multi-locus VNTR analysis (MLVA) profile being completed in chromosomal order, which was cgd1, cgd4, MSF, cgd5, cgd6, cgd8 and MM19. Care was taken when examining the peaks to determine the size of the fragment. Wide and low peaks, stutter peaks and peaks bleeding across fluorescent channels were disregarded as true peaks. If no peak appeared, the corresponding fragment was defined as a null allele (labelled as Ø) and mixed alleles were only identified if the peak height ratio between the two peaks was greater than 0.25.

For each allele, representative samples from both adults and calves were sent for sequence analysis. The numbers of sequence repeats were manually counted, and the information was compared against the size fragment analysis data to ensure the results being reported were accurate (see Supplementary Table S1).

### 2.11 Statistical analysis

Statistical analyses (odds ratios, Fisherʼs exact tests and two-way Chi-square tests) were performed using MedCalc for Windows, version 19.4 (MedCalc Software, Ostend, Belgium) (Campbell, 2007; Richardson, 2011; Parshall, 2013).

## 3 Results

### 3.1 Species of *Cryptosporidium* found in adult cattle

A total of 224 faecal samples were collected from the adult cattle over the first seven weeks of the calving session, from both pre-partum dams (Pen A, *n* = 112; mean *n* = 16; weekly range 12–21) and post-partum dams (Pen B, *n* = 112; mean *n* = 19; weekly range 14–24). One cow gave birth while still housed in Pen A. Following the birth, the dam was moved to Pen B and the calf was moved to Pen CA.

#### 3.1.1 Pre-partum cattle

In Pen A, 31.3% (35/112) of the faecal samples tested positive for the presence of *Cryptosporidium* spp. DNA. Positive samples were seen from Week 2 after the beginning of the calving season (see Table 2), where samples positive for *C. parvum* (*n* = 1) and *C. bovis* (*n* = 1) were identified. In Week 3, *C. andersoni* was identified in Pen A in two positive samples. The highest number of positive samples was observed in Pen A during Week 4, where 77.8% (14/18) of the samples were found to contain *Cryptosporidium* DNA (all *C. parvum-*positive), while in Weeks 5, 6 and 7, 7/16, 5/16 and 5/21 positive samples were identified, respectively (5 *C. parvum-*positive samples in each week).

**Table 2:** Species identification of *Cryptosporidium*-positive samples detected in pre- and post-partum adult cattle.

Week	Pen	Samples collected (*n*)	Negative (*n*)	Positive (*n*)	*C. parvum* (*n*)	*C. bovis* (*n*)	*C. andersoni* (*n*)

1	A	14	14	0	–	–	–
	B	–	–	–	–	–	–
2	A	15	13	2	1	1	–
	B	21	19	2	1	–	1
3	A	12	10	2	–	–	2
	B	24	21	3^a^	1	2	1
4	A	18	4	14	14	–	–
	B	14	4	10	10	–	–
5	A	16	9	7^a^	5	–	3
	B	16	11	5	4	–	1
6	A	16	11	5^a^	5	1	2
	B	16	12	4^a^	4	–	1
7	A	21	16	5	5	–	–
	B	21	11	10^a^	10	–	6

a Samples contained more than one *Cryptosporidium* spp.

In Pen A, adult cattle were 49 times (odds ratio, OR: 49.00, 95% confidence interval, CI: 4.85–495.23, *P* = 0.001) more likely to test *C. parvum*-positive during Week 4 of calving (14/18 *C. parvum*-positive samples), compared to Week 2 when the first *C. parvum*-positive sample (1/14) was identified (Table 2). The statistical significance of this finding was confirmed by using Fisherʼs exact test (*P* < 0.0001). The adults in Pen A continued to have increased odds of shedding *C. parvum* at Weeks 5, 6 and 7 (OR 6.36, 3.23 and 4.37, respectively) compared to Week 2; however, this risk was not considered statistically significant (*P* = 0.135, *P* = 0.346 and *P* = 0.222, respectively).

#### 3.1.2 Post-partum cattle

In Pen B, 30.4% (34/112) of the faecal samples tested positive for *Cryptosporidium* spp. DNA, with *C. parvum* DNA-positive samples being identified on at least one occasion each week where samples were collected (i.e. calving season Weeks 2–7). The highest number of positive samples in Pen B was observed during Week 4 of calving, where 71.4% (10/14) of the samples tested positive (Table 2).

At Week 4 of the calving season, the animals in Pen B had a statistically significantly higher risk of shedding *C. parvum*, compared to when the first *C. parvum*-positive samples were identified in this pen at Week 2 (OR: 50.00, 95% CI: 4.92–508.33; Fisherʼs exact test *P* < 0.0001). Compared to Week 2 (first *C. parvum-*positive sample), the likelihood of testing *C. parvum-*positive continued to remain elevated at Weeks 5, 6 and 7 (OR: 9.10, 6.67 and 18.18, respectively). The risk had a borderline significance on Week 5 (*P* = 0.056) and was significant again on Week 7 (*P* = 0.009).

There was no significant difference in the likelihood of being *C. parvum-*positive when comparing pre- and post-natal cattle (Pen A and Pen B) (OR: 1.03, 95% CI: 0.55–1.93, *P* = 0.922).

Representative sequences of the 18S rRNA gene fragments for *C. andersoni* and *C. bovis* were submitted to the GenBank database under the accession numbers OR593321-OR593323.

### 3.2 Clinical observations

Between weeks 5 and 19 of the calving season, evidence of diarrhoea was repeatedly observed in the neonatal animals across most pens. Animals, which showed clear physical evidence of diarrhoea, were generally the youngest animals in each pen and as the calves became older, the incidence of diarrhoea tended to decrease. Along with diarrhoea, the calves also demonstrated other common clinical signs of cryptosporidiosis; these included a general depression in their demeanour along with signs of listlessness and a reluctance to stand. In some severe cases, calves also demonstrated a reluctance to feed, resulting in the animals needing to be bottle-fed by the stockmen. In very severe cases veterinary intervention was required and the calves received fluids intravenously. There was no evidence of clinical cryptosporidiosis amongst the adult animals during the study period.

### 3.3 Rates of calf mortality

The numbers of calves that were born alive or dead and that died before 42 days of age during the 2020–2021, 2021–2022 and 2022–2023 calving seasons are provided in Table 3. The number of calves being born alive each year is relatively consistent (252, 242 and 241 respectively). Although the number of calves being born dead each year were much higher in the 2020–2021 (*n* = 20) season compared to 2021–2022 and 2022–2023 where only (*n* = 9 and *n* = 14, respectively) died at birth, these differences were not significant (Fisherʼs exact test, *P* = 0.084 and *P* = 0.479, respectively). When levels of neonatal calf mortality were compared, the highest numbers of calves that died during the first 42 days after birth was recorded during our main study season (2021–2022), with 7.9% (19/242) compared to 3.2% (8/252) during 2020–2021 and 0.8% (2/241) during 2022–2023. These differences in neonatal calf mortality were statistically significant in both comparisons (*P* = 0.046 and *P* = 0.0002, respectively). It is also interesting to note that the majority (9/19) of the calf deaths were observed between Weeks 11–15 of the calving season. Although the mean age (in days) when neonatal deaths occurred was comparable across the three seasons being 15.4, 16.0 and 12.5 days respectively, the range for the 2021–2022 season was wider and included calves from 2 to 28 days of age, while the range for the other seasons ranged was 7–19 days and 9–16 days, respectively.

**Table 3:** Number of calves born alive or dead and number of calves that died before 42 days of age.

Calving season (years)	2020–2021	2021–2022	2022–2023

Calves born alive (*n*)	252	242	241
Calves born dead (*n*)	20	9	14
Calves died before 42 days (*n*)	8	19	2
Mean age at death (days)	15.4	16	12.5
Age range (days)	7–19	2–28	9–16

### 3.4 *Cryptosporidium parvum* in calf samples

**Fig. 1:**
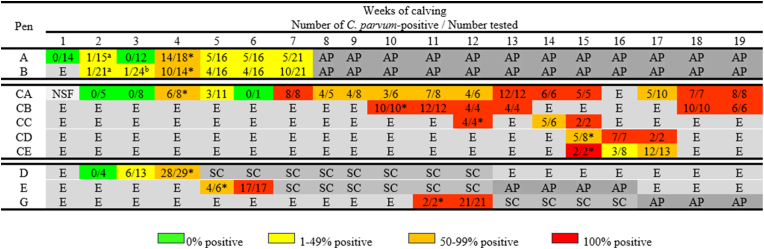
Numbers of *C. parvum*-positive samples in adult cattle and calves throughout the beginning of a calving season. *Notes*: ^a^*C*. *parvum gp60* genotype IIaA15R1 identified; ^b^*C*. *parvum gp60* genotype IIaA14G2R1 identified; *C. *parvum gp60* genotype IIaA15G2R1 first identified (this is the only *gp60* genotype identified from that point onwards). *Abbreviations*: AP, animals in pen, but samples were not collected; E, pen empty (no animals); NSF, animals in pen, but no samples were found; SC, samples collected but not analysed.

A total of 312 calf samples were collected and tested across the first 19 weeks of the calving season (see Fig. 1). Positive results with the *C. parvum* 18S PCR primers were found in 79.5% (248/312) of the samples tested.

In the calves, the first *C. parvum-*positive samples were detected in Pen D at Week 3 of calving (Fig. 1), where 46.2% (6/13) of the samples tested positive. However, by Week 4 in Pen D, 96.6% (28/29) of the samples were *C. parvum-*positive, which meant that the calves were 32 times (OR: 32.67, 95% CI: 3.36–317.24) more likely to test positive for *Cryptosporidium* in the space of a week (Fisherʼs exact test, *P* = 0.00043).

Samples from Pen CA tested PCR-positive for *C. parvum* from Week 4 onwards (at least once per week) in every week of the calving season, except for Week 6; however, during this week only one sample was collected from this pen. Every sample collected from Pen CA tested PCR-positive during 6 out of 17 weeks of the study (Fig. 1). Overall, 67.2% (82/122) of the samples collected from Pen CA tested PCR-positive for *C. parvum*.

Pen CB was used for the first-time during Week 10 of calving, where it immediately tested positive and on all subsequent times thereafter, every sample (100%; 46/46) collected from Pen CB tested PCR-positive for *C. parvum*.

Pen CC was only used for three weeks between the calving weeks 12–15. Samples from Pen CC tested positive continuously from the first week of use; during these sampling points 91.7% (11/12) of the samples were PCR-positive for *C. parvum* and this is another example where animals are testing positive directly on being introduced into a pen.

Pens CD and CE were unusual, in that they had not been used to house calves in several calving seasons and were used during this calving season to allow the main neonatal Pens CA and CB to be cleaned and disinfected. Even though no cattle had been housed in Pens CD and CE for several years, 82.6% (14/17) and 73.9% (17/23) of the samples tested PCR-positive for *C. parvum*, respectively. Both Pens CD and CE had positive samples from the very first sampling point and demonstrated at least one week each where every sample tested positive.

Samples from calves in Pens D, E and G were only analysed for a short period of time, as almost all the samples from each pen tested positive for the presence of *C. parvum* within one to three weeks of calves being placed in the pens. For Pen D, 96.6% (28/29) of the samples were positive in the third week of use. In Pen E, all (17/17) samples tested PCR-positive on the second week of use and in Pen G every sample collected (23/23) was PCR-positive from its first week of use.

**Fig. 2:**
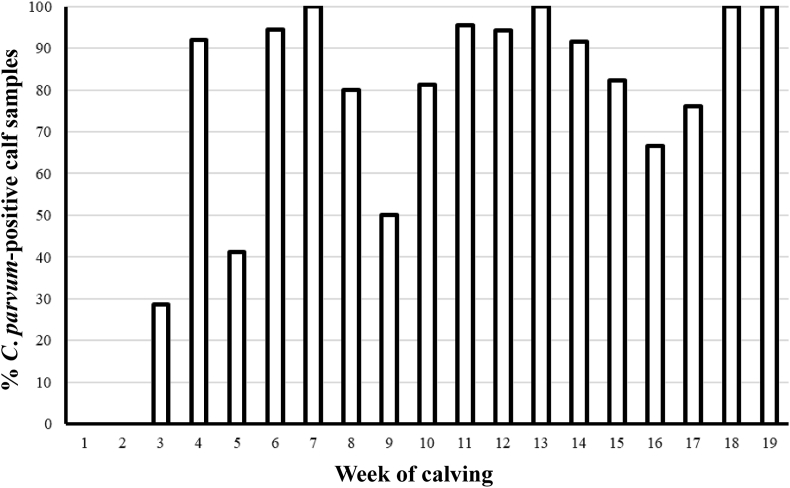
Percentage of *C. parvum-*positive calf samples over the first 19 weeks of the calving season.

A comparison of the overall percent of positive calf samples between Weeks 1–19 is illustrated in Fig. 2 and shows that the overall percentage of positive calf samples increases rapidly between Week 3 (28.6%) and Week 4 (91.9%) and remains elevated for the remainder of the calving season, meaning a significantly higher proportion of positive calves in Week 4 compared to Week 3 (*P* = 0.0001) (Fig. 2). From Week 4 onwards an average of 84.1% of calf samples were positive (range 41.2–100%).

### 3.5 Comparison of infection rates with *C. parvum* in adult cattle and calves

A comparison of the likelihood of being *C. parvum* infected between the adult cattle and calves, over the first seven weeks of calving (where comparable samples were available) revealed that calves were 10.84 times more likely to be *C. parvum*-positive (OR: 10.84, 95% CI: 7.23–16.25, *P* ≤ 0.0001) than adult cattle.

A “N-1” *χ*^2^ test was applied to compare the proportion of positive adult cattle and calves throughout the first seven weeks of the calving period, where comparable data were available. Overall, 23.7% (53/224) of the adult samples tested *C. parvum-*positive; this is significantly lower (*P* ≤ 0.0001) compared to 65.4% (72/110) of the calf samples collected during the first seven weeks.

### 3.6 Genotypic variation of the *C. parvum*-positive adult samples

#### 3.6.1 Pre-partum cattle

In Pen A, *gp60* genotypes could be determined for 20 of the *C. parvum-*positive adult samples. One sample from Week 2 had a IIaA15R1 genotype, and the remaining 19 samples, were all identified as IIaA15G2R1. Complete MLVA profiles were generated for 13 out of 19 samples with the IIaA15G2R1 *gp60* genotype, all of which had the MLVA profile of 4-14-5-8-18-37-16 (see Supplementary Table S2A). Unfortunately, the IIaA15R1 sample from Pen A failed to produce a complete MLVA profile (Ø-Ø-Ø-8-Ø-Ø-Ø), even after repeated testing (see Supplementary Table S2A).

#### 3.6.2 Post-partum cattle

In Pen B, the first two *C. parvum-*positive samples identified (Weeks 2 and 3) were IIaA15R1 and IIaA14G2R1, respectively (see Fig. 1). The remaining 16 samples with identifiable *gp60* genotypes from Week 4 onwards were all IIaA15G2R1. Examination of the MLVA profiles from Pen B revealed that the IIaA15R1-positive sample had an incomplete profile (4-14-5-8-18-Ø-14) compared to the 4-14-5-8-18-37-16 profile produced by the IIaA15G2R1-positive samples from Weeks 4 and 6 of the calving season. Even with a null peak for the cgd8 allele, the results demonstrated a clear difference at MM19, where 14 repeat sequences were seen, compared to the 16 repeats observed in the IIaA15G2R1. Unfortunately, the IIaA14G2R1-positive sample (from Week 3) failed to produce a readable MLVA profile (Ø-Ø-Ø-Ø-Ø-Ø-Ø), even after repeated attempts (see Supplementary Table S2B).

### 3.7 Genotypic variation of the *C. parvum*-positive calf samples

For the calves, 125 samples returned readable *gp60* genotypes. All the calf samples (125/125) collected from Pens CA-CE, D, E and G were identified as IIaA15G2R1. Additionally, 31 samples (Pens CA-CE, D, E and G) also returned complete MLVA profiles of 4-14-5-8-18-37-16, which was identical to that seen in the adults in Pens A and B. Identical *gp60* and MLVA profiles were observed in the calf samples, from all the pens in samples from four weeks of calving onwards (see Supplementary Tables S2C–2J). The remaining calf samples produced incomplete MLVA profiles. Although the profiles of many of the calves were incomplete, no unique genotypes were identified in any of the calf samples.

Representative sequences of the *gp60* gene fragments from the different *C. parvum* genotypes were submitted to the GenBank database under the accession numbers (OR590769-OR590782).

### 3.8 Comparison of efficiency of VNTR PCRs

**Fig. 3:**
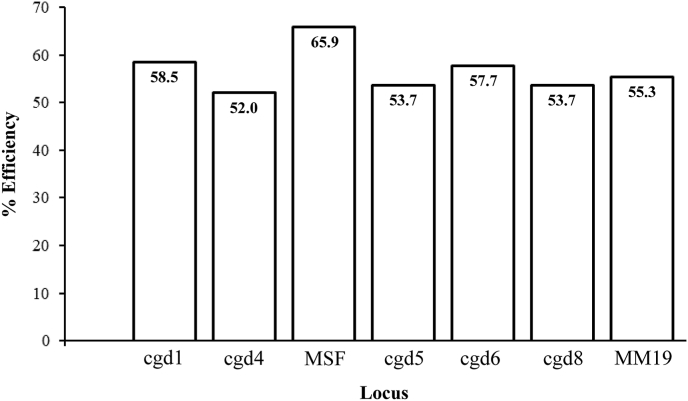
Efficiency of individual Variable Number Tandem Repeat (VNTR) PCRs. Results from the calf samples, illustrating the percentage of reactions that generated clearly identifiable peaks for each of the VNTR loci.

The efficiency of each of the VNTR PCR primer pairs was compared using a combination of *C. parvum-*positive adult and calf samples (*n* = 123) that had a readable result for at least one allele. The most efficient VNTR PCR primers amplified the locus MSF, which had positive results for 65.9% (81/123) of the samples tested. The least efficient PCR primers amplified cdg4 with only 64 out of 123 (52.0%) positive results (Fig. 3). The remaining PCRs for cdg1, cdg5, cdg6 cdg8 and MM19 were comparable in their efficiency (ranging between 53.7 and 58.5%). When the efficiencies of the MSF and cdg4 PCR were compared using a Fisherʼs exact test, MSF was found to be significantly more efficient than cdg4 (*P* = 0.038). No other statistical differences were observed when comparing the other PCR.

## 4 Discussion

To our knowledge, this is the first longitudinal study of dairy cattle to examine faecal samples from adult cattle before (pre-partum) and at the beginning of a calving season (post-partum), as well as sampling neonatal calves from very early stages of the calving season for the presence of *Cryptosporidium*. The results from this study clearly demonstrate that infections with *Cryptosporidium* spp. are common among both pre- and post-partum cattle and that infections with *C. parvum* are highly prevalent amongst neonatal dairy calves, with infections occurring at a very early age.

In this study, 30.8% (69/224) of the samples collected from the adult cattle tested positive for *Cryptosporidium* spp. *Cryptosporidium parvum* was identified in 76.8% (53/69) of the positive samples, either as a single infection, or in co-infections with both *C. andersoni* and *C. bovis*. These findings are consistent with previous longitudinal studies on this farm where 27.3% of the adult samples tested *Cryptosporidium*-positive (Thomson et al., 2019b). Also, in another study *C. parvum* was the most commonly recognised species of *Cryptosporidium* in adult dairy cattle in Belgium (70%) and the Netherlands (75%) (Pinto et al., 2021). Cryptosporidiosis is known to be more of an issue with dairy cattle than beef cattle. Previous studies have demonstrated that dairy cattle (particularly calves) posed a greater risk to human health than beef cattle/calves (OʼHandley, 2007; Dixon et al., 2011). This is likely a farm management issue, with dairy cattle and their calves being housed indoors for extended periods of time (i.e. over winter).

Possible *C. parvum* transmission routes can be inferred from when calves became infected. Calf-to-calf transmission would seem to be one of the major routes of infection, due to the synchronicity of infection between calf pens. The most probable scenario would be that an infected calf in pen CA was transferred to pen D, thus spreading infection. The lack of variation in all the calf pens suggest that the IIaA15G2R1 population of *C. parvum* was fully established in pen CA very early in the calving season. Once the infection became established, it spread throughout the farm with relative ease. Other transmission methods, which can be proposed, are as follows. First, the diarrhoeic nature of the faeces from infected calves in pen CA could spread oocysts to pens CB-CC due to their proximity to each other. Secondly, *C. parvum* oocysts from previous calving seasons could have persisted in the pens causing infection. Thirdly, the stockmen themselves could be a route of transmission through contaminated PPE, as they work with both infected and uninfected animals and will most likely get faeces containing *C. parvum* oocysts onto their overalls. Finally, the adult cattle could be the source of infection.

Where the initial infection in calves comes from at the beginning of the calving season is still unclear, as though both the pre- and post-partum adult cattle tested *C. parvum-*positive during Week 2 of the calving season, the genotype that was identified (IIaA15R1) was never detected in any of the neonatal calf samples of any age. The only genotype identified in the calves was IIaA15G2R1. The first calf samples to test *C. parvum-*positive were isolated from Pen D during the third week of the season, where 46.1% (6/13) of the samples were *C. parvum-*positive. Unfortunately, we were unable to identify the *gp60* genotypes of any of these samples even after repeated testing; this may have been due to low numbers of oocysts in the samples at the early stages of calving/infection. However, it is highly likely that these samples were the same IIaA15G2R1 genotype that was identified in all the other calf samples (*n* = 7) at the following sampling point three days later and was also highly prevalent amongst the adult cattle during Week 4 of calving.

There are many factors that influence the spread of *C. parvum* amongst neonatal farm animals. One of importance is the high rates of oocyst shedding in neonatal dairy calves. Several studies have calculated the numbers of oocysts shed by young calves. A study by Nydam et al. (2001) demonstrated that a single naturally infected 6-day-old dairy calf could produce 3.89 × 10^10^ *C. parvum* oocysts by the age of 12 days. Additionally, dairy calves can shed oocysts for 14–21 days (Zambriski et al., 2013), highlighting the potential scale of the problem faced by dairy farmers in trying to control the spread of cryptosporidiosis amongst young animals. The situation is further complicated by the low infectious dose of *C. parvum*, meaning that infections can become quickly established in calf pens. In experimentally infected neonatal cattle, an estimated 16.6 oocysts were associated with parasite shedding and diarrhoea in 50% of infected calves (Zambriski et al., 2013), while in healthy human volunteers, 30–1000 oocysts (median 132 oocysts) caused infection and oocyst shedding (DuPont et al., 1995) again highlighting the danger that *C. parvum* poses not only to animals but also to human health.

At the beginning of the calving season, the infecting doses were clearly relatively low, as there were only a few positive samples amongst the young animals over the first few weeks of calving and the animals appeared healthy (non-diarrhoeic). Even those samples that were *C. parvum-*positive did not return positive *gp60* results, which is suggestive of low numbers of oocysts, as *gp60* is a single copy gene (Strong et al., 2000), meaning many more oocysts are required to produce a positive PCR result, than are required for amplification of the multi-copy 18S rRNA gene (where each oocyst contains 20 copies) (Le Blancq et al., 1997). However, as the calving season progressed, the number of oocysts in the environment would have increased quickly. An increasing number of oocysts in the environment means not only an increased chance of exposure, but as the infective dose of oocysts increase then it is also likely that the pre-patent period before a calf became diarrhoeic would decrease. The duration each calf shed oocysts for could also increase, as has previously been shown in experimentally infected calves (Zambriski et al., 2013).

The rates of neonatal calf mortality were significantly higher in the 2021–2022 season compared to other seasons. Although we know from the numbers of positive samples that levels of infection were high, we do not know how many of these deaths were as a consequence of cryptosporidiosis or other pathogens and whether the change in dominant genotype present in the calf population also played a role in these deaths. Recent experimental data from lambs clearly demonstrated differences in the pathogenicity of isolates of *C. parvum*, where the IIaA15G2R1 genotype was more virulent than the IIaA19G2R1 (originally isolated from this study farm), in terms of severity of clinical symptoms and numbers of oocysts shed by infected animals (Bartley et al., 2023). Clinical observations from the present study also show that the animals that were diarrhoeic also generally demonstrated other clinical symptoms associated with an infection with *C. parvum*.

Historically the study farm demonstrated greater genetic diversity of *gp60* amongst the adult cattle as well as a completely different genotype predominating in the calf population. In a previous study, the IIaA19G2R1 genotype was identified in human cases associated with veterinary students handling calves on the farm as well as within the calf population (Gait et al., 2008). In a previous study Thomson et al. (2019a) identified only the IIaA19G2R1 genotype in all calves ≤ 6 weeks of age, during calving seasons over two years. At the same time four different *gp60* genotypes, IIaA15R1, IIaA15G2R1, IIaA18G2R1 and IIaA19G2R1, were routinely identified in adult cattle (Thomson et al., 2019b).

During the present study, calf (≤ 6 weeks-old) samples were tested and typed that were collected during the 2019–2020 calving season (two years before the main study). Positives amongst these were identified as the IIaA19G2R1 (4-14-5-7-27-32-19) genotype. Calf (< 6 weeks-old) samples, which were collected during the 2022–2023 calving season (the year after the main study), were identified as IIaA15G2R1, but failed to produce readable VNTR profiles. The historical and the new genotype data collected on this farm indicates that the IIaA19G2R1 genotype that had been stably maintained within the calf population on the farm for over a decade was replaced sometime between 2020 and 2021 with the IIaA15G2R1 genotype, which is still present in the calves in 2023. It is unclear if the IIaA15G2R1 is more pathogenic than the IIaA19G2R1, or if it is somehow just better adapted to calves and therefore more able to establish infections. Recently published data from lambs experimentally challenged with two distinct isolates of *C. parvum* clearly demonstrated that there are differences in pathogenicity between isolates of *C. parvum*, which also affected oocyst shedding profiles (Bartley et al., 2023). Another reason for the increased fecundity of the IIaA15G2R1 genotype may be due to increased levels of the viral symbiont *Cryptosporidium parvum virus* (CPV), which has been hypothesised to be linked to parasite virulence, though this would need to be further investigated (Jenkins et al., 2008).

As we only collected environmental samples and did not sample per rectum, we cannot definitively state how many *C. parvum-*infected neonatal calves were actively shedding oocysts on the farm. However, this number is likely to be very high, as on 16 separate occasions, every sample collected from an individual calf pen tested positive for *C. parvum*. Except for Pen D, where a maximum of 96.5% (27/28) of the samples were positive, every other calf pen had at least one occasion where all samples were positive. For some of the pens every sample tested positive for several weeks in a row and for Pen CB every sample collected (46/46) tested positive for *C. parvum* (see Fig. 1).

## 5 Conclusions

This study demonstrated that there were comparable levels of *C. parvum* infections in pre- and post-partum dairy cattle at the beginning of a calving season. Comparatively, there were significantly higher levels of *C. parvum* infection in neonatal calves than in the adult cattle. This study also demonstrated that the time taken for calves to become infected decreases as the calving season progresses, with almost all samples testing positive from about week six of calving onwards. After Week 4 of calving, only a single genotype of *C. parvum* (IIaA15G2R1) was found in the adults, while IIaA15G2R1 was the only genotype of *C. parvum* identified in the calves.

## Funding

The work was supported by the 10.13039/100012095Scottish Government through the 10.13039/100011310Rural and Environment Science and Analytical Services (RESAS) Strategic Research Programme 2022–2027, project number MRI-A2-9: “The role of wildlife and livestock in the emergence and persistence of zoonosis in Scotland, and novel interventions”.

## Ethical approval

No ethical approval was required for this study.

## CRediT authorship contribution statement

**Paul M. Bartley:** Conceptualization, Methodology, Formal analysis, Writing – original draft, Writing – review & editing, Visualization. **Johan H. Standar:** Methodology, Formal analysis, Writing – original draft, Writing – review & editing, Visualization. **Frank Katzer:** Conceptualization, Methodology, Formal analysis, Writing – original draft, Writing – review & editing, Funding acquisition, Visualization, Project administration. All authors read and approved the final manuscript.

## Declaration of competing interests

The authors declare that they have no known competing financial interests or personal relationships that could have appeared to influence the work reported in this paper. Given their role as Co-Editor, Frank Katzer had no involvement in the peer review of this article and has no access to information regarding its peer review. Full responsibility for the editorial process for this article was delegated to Editor-in-Chief Professor Aneta Kostadinova.

## Data Availability

The data supporting the conclusions of this article are included within the article and its supplementary files. Representative sequences of the 18S rRNA gene fragments for *C. andersoni* and *C. bovis* were submitted to the GenBank database under the numbers OR593321-OR593323. Representative sequences of the *gp60* gene fragments from the different *Cryptosporidium* *parvum* genotypes were submitted to the GenBank database under the accession numbers (OR590769-OR590782).

## References

[bib1] Atwill E.R., Pereira M.G.C. (2003). Lack of detectable shedding of *Cryptosporidium parvum* oocysts by periparturient dairy cattle. J. Parasitol..

[bib2] Bartley P.M., Thomson S., Jonsson N.N., Taroda A., Elisabeth A.I., Katzer F. (2023). Differences in virulence and oocyst shedding profiles in lambs experimentally infected with different isolates of *Cryptosporidium parvum*. Curr. Res. Parasitol. Vector Borne Dis..

[bib3] Brook E.J., Anthony Hart C., French N.P., Christley R.M. (2009). Molecular epidemiology of *Cryptosporidium* subtypes in cattle in England. Vet. J..

[bib4] Campbell I. (2007). Chi-squared and Fisher-Irwin tests of two-by-two tables with small sample recommendations. Stat. Med..

[bib5] Castro-Hermida J.A., González-Losada Y.A., Mezo-Menéndez M., Ares-Mazás E. (2002). A study of cryptosporidiosis in a cohort of neonatal calves. Vet. Parasitol..

[bib6] Chalmers R.M., Robinson G., Elwin K., Elson R. (2019). Analysis of the *Cryptosporidium* spp. and *gp60* subtypes linked to human outbreaks of cryptosporidiosis in England and Wales, 2009 to 2017. Parasites Vectors.

[bib7] Dixon B., Parrington L., Cook A., Pintar K., Pollari F., Kelton D., Farber J. (2011). The potential for zoonotic transmission of *Giardia duodenalis* and *Cryptosporidium* spp. from beef and dairy cattle in Ontario, Canada. Vet. Parasitol..

[bib8] DuPont H.L., Chappell C.L., Sterling C.R., Okhuysen P.C., Rose J.B., Jakubowski W. (1995). The infectivity of *Cryptosporidium parvum* in healthy volunteers. N. Engl. J. Med..

[bib9] Gait R., Soutar R.H., Hanson M., Fraser C., Chalmers R. (2008). Outbreak of cryptosporidiosis among veterinary students. Vet. Rec..

[bib10] Helmy Y.A., von Samson-Himmelstjerna G., Nöckler K., Zessin K.-H. (2015). Frequencies and spatial distributions of *Cryptosporidium* in livestock animals and children in the Ismailia province of Egypt. Epidemiol. Infect..

[bib11] Huang J., Chen M., He Y., Chen H., Huang M., Li N. (2023). *Cryptosporidium equi* n. sp. (Apicomplexa: Cryptosporidiidae): Biological and genetic characterisations. Int. J. Parasitol..

[bib12] Jenkins M.C., Higgins J., Abrahante J.E., Kniel K.E., OʼBrien C., Trout J. (2008). Fecundity of *Cryptosporidium parvum* is correlated with intracellular levels of the viral symbiont CPV. Int. J. Parasitol..

[bib13] Le Blancq S.M., Khramtsov N.V., Zamani F., Upton S.J., Wu T.W. (1997). Ribosomal RNA gene organization in *Cryptosporidium parvum*. Mol. Biochem. Parasitol..

[bib14] Nydam D.V., Wade S.E., Schaaf S.L., Mohammed H.O. (2001). Number of *Cryptosporidium parvum* oocysts or *Giardia* spp. cysts shed by dairy calves after natural infection. Am. J. Vet. Res..

[bib15] OʼHandley R.M. (2007). *Cryptosporidium parvum* infection in cattle: Are current perceptions accurate?. Trends Parasitol..

[bib16] Parshall M.B. (2013). Unpacking the 2 × 2 table. Heart Lung.

[bib17] Pinto P., Ribeiro C.A., Hoque S., Hammouma O., Leruste H., Detriche S. (2021). Cross-border investigations on the prevalence and transmission dynamics of *Cryptosporidium* species in dairy cattle farms in western mainland Europe. Microorganisms.

[bib18] Prediger J., Ježková J., Holubová N., Sak B., Konečný R., Rost M. (2021). *Cryptosporidium sciurinum* n. sp. (Apicomplexa: Cryptosporidiidae) in Eurasian red squirrels (*Sciurus vulgaris*). Microorganisms.

[bib19] Richardson J.T. (2011). The analysis of 2 × 2 contingency tables - yet again. Stat. Med..

[bib20] Robinson G., Perez-Cordon G., Hamilton C., Katzer F., Connelly L., Alexander C.L., Chalmers R.M. (2022). Validation of a multilocus genotyping scheme for subtyping *Cryptosporidium parvum* for epidemiological purposes. Food Waterborne Parasitol..

[bib21] Ryan U.M., Feng Y., Fayer R., Xiao L. (2021). Taxonomy and molecular epidemiology of *Cryptosporidium* and *Giardia* - a 50-year perspective (1971–2021). Int. J. Parasitol..

[bib22] Shaw H.J., Armstrong C., Uttley K., Morrison L.J., Innes E.A., Katzer F. (2021). Genetic diversity and shedding profiles for *Cryptosporidium parvum* in adult cattle and their calves. Curr. Res. Parasitol. Vector Borne Dis..

[bib23] Strong W.B., Gut J., Nelson R.G. (2000). Cloning and sequence analysis of a highly polymorphic *Cryptosporidium parvum* gene encoding a 60-kilodalton glycoprotein and characterization of its 15- and 45-kilodalton zoite surface antigen products. Infect. Immun..

[bib24] Thomson S., Hamilton C.A., Hope J.C., Katzer F., Mabbott N.A., Morrison L.J., Innes E.A. (2017). Bovine cryptosporidiosis: Impact, host-parasite interaction and control strategies. Vet. Res..

[bib25] Thomson S., Innes E.A., Jonsson N.N., Katzer F. (2019). Shedding of *Cryptosporidium* in calves and dams: evidence of re-infection and shedding of different *gp60* subtypes. Parasitology.

[bib26] Thomson S., Innes E.A., Jonsson N.N., Katzer F. (2019). A multiplex PCR test to identify four common cattle-adapted *Cryptosporidium* species - Corrigendum. Parasitol. Open.

[bib27] Tůmová L., Ježková J., Prediger J., Holubová N., Sak B., Konečný R. (2023). *Cryptosporidium mortiferum* n. sp. (Apicomplexa: Cryptosporidiidae), the species causing lethal cryptosporidiosis in Eurasian red squirrels (*Sciurus vulgaris*). Parasites Vectors.

[bib28] Wang R., Ma G., Zhao J., Lu Q., Wang H., Zhang L. (2011). *Cryptosporidium andersoni* is the predominant species in post-weaned and adult dairy cattle in China. Parasitol. Int..

[bib29] Wells B., Thomson S., Ensor H., Innes E.A., Katzer F. (2016). Development of a sensitive method to extract and detect low numbers of *Cryptosporidium* oocysts from adult cattle faecal samples. Vet. Parasitol..

[bib30] Zambriski J.A., Nydam D.V., Wilcox Z.J., Bowman D.D., Mohammed H.O., Liotta J.L. (2013). *Cryptosporidium parvum*: determination of ID(5)(0) and the dose-response relationship in experimentally challenged dairy calves. Vet. Parasitol..

