# Supplementary material for: Genetic characterisation of Cryptosporidium parvum in dairy cattle and calves during the early stages of a calving season
Source: Curr Res Parasitol Vector Borne Dis. 2023 Dec 1;5:100160. doi: 10.1016/j.crpvbd.2023.100160 (PMC10727939; doi:10.1016/j.crpvbd.2023.100160)
Supplement: Multimedia component 1 [file mmc1.pdf]

Supplementary Figure S1

Schematic diagram of the building and pen layout of the dairy farm (distances are not to scale)

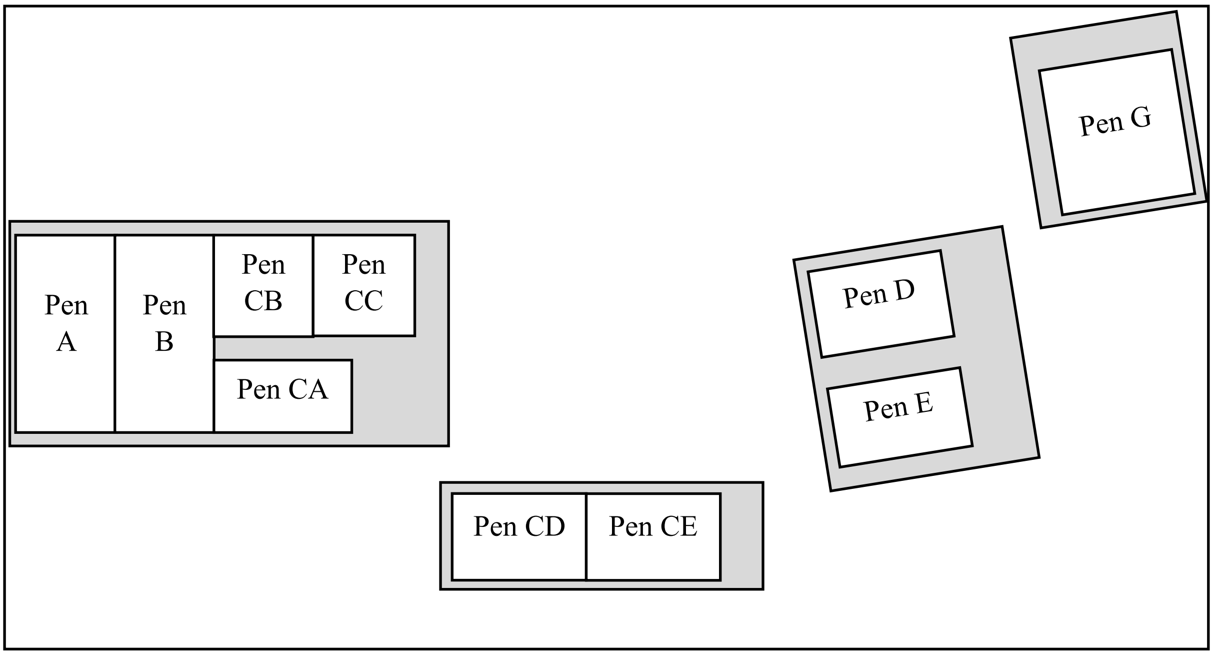

Pen A	Pre-partum dams in the last 1–2 weeks of pregnancy
Pen B	Post-partum dams (calves for first 12 h after birth)
Pens CA-CE	Calves for days 1–7 days after birth (aim of 2–3 days only) (maximum 7 calves per pen).
Pens D, E and G	Calves from ~1 week to 10 weeks of age
