# Supplementary material for: Genetic characterisation of Cryptosporidium parvum in dairy cattle and calves during the early stages of a calving season
Source: Curr Res Parasitol Vector Borne Dis. 2023 Dec 1;5:100160. doi: 10.1016/j.crpvbd.2023.100160 (PMC10727939; doi:10.1016/j.crpvbd.2023.100160)
Supplement: Multimedia component 2 [file mmc2.pdf]

**Supplementary Table S1.** Fragment sizes (bp) / numbers of repeat regions and overall VNTR profiles of *C. parvum*-positive samples from both pre-partum adult cattle and calves.

| **Sample** | **Loci Fragment size / Number of repeats** | | | | | | | **Final genotype** |
|---|---|---|---|---|---|---|---|---|
| | **cgd1** | **cgd4** | **MSF** | **cgd5** | **cgd6** | **cgd8** | **MM19** | |
| A49 | 129 / 4 | 267 / 14 | 149 / 5 | 98 / 8 | 132 / 18 | 301 / 37 | 166 / 16 | 4-14-5-8-18-37-16 |
| A50 | 129 / 4 | 267 / 14 | 149 / 5 | 98 / 8 | 132 / 18 | 301 / 37 | 166 / 16 | 4-14-5-8-18-37-16 |
| CA91 | 129 / 4 | 267 / 14 | 149 / 5 | 98 /8 | 132 / 18 | 301 / 37 | 166 / 16 | 4-14-5-8-18-37-16 |
| CB1 | 129 / 4 | 267 / 14 | 149 / 5 | 98 / 8 | 132 / 18 | 301 / 37 | 166 / 16 | 4-14-5-8-18-37-16 |
| CC1 | 129 / 4 | 267 / 14 | 149 / 5 | 98 / 8 | 132 / 18 | 301 / 37 | 166 / 16 | 4-14-5-8-18-37-16 |
| CC11 | 129 / 4 | 267 / 14 | 149 / 5 | 98 / 8 | 132 / 18 | 301 / 37 | 166 / 16 | 4-14-5-8-18-37-16 |
| CC12 | 129 / 4 | 267 / 14 | 149 / 5 | 98 / 8 | 132 / 18 | 301 / 37 | 166 / 16 | 4-14-5-8-18-37-16 |
| G34 | 129 / 4 | 267 / 14 | 149 / 5 | 98 / 8 | 132 / 18 | 301 / 37 | 166 / 16 | 4-14-5-8-18-37-16 |
