# Supplementary material for: Genetic characterisation of Cryptosporidium parvum in dairy cattle and calves during the early stages of a calving season
Source: Curr Res Parasitol Vector Borne Dis. 2023 Dec 1;5:100160. doi: 10.1016/j.crpvbd.2023.100160 (PMC10727939; doi:10.1016/j.crpvbd.2023.100160)
Supplement: Multimedia component 3 [file mmc3.pdf]

Supplementary Table S2A. MLVA profiles of pre-partum cattle in Pen A.

Sample ID	Sample Point	Gp60 genotype	Loci / Number of repeats							Number of Loci	Final genotype
			cgd1	cgd4	MSF	cgd5	cgd6	cgd8	MM19		
**A26**	2-1	IIaA15R1	Ø	Ø	Ø	8	Ø	Ø	Ø	1	Ø-Ø-Ø-8-Ø-Ø-Ø
**A47**	4-1	IIaA15G2R1	Ø	Ø	5	8	18	Ø	Ø	3	Ø-Ø-5-8-18-Ø-Ø
**A48**	4-1	IIaA15G2R1	4	14	5	8	18	37	16	7	4-14-5-8-18-37-16
**A49**	4-1	IIaA15G2R1	4	14	5	8	18	37	16	7	4-14-5-8-18-37-16
**A50**	4-1	IIaA15G2R1	4	14	5	8	18	37	16	7	4-14-5-8-18-37-16
**A51**	4-1	IIaA15G2R1	4	Ø	5	Ø	Ø	Ø	Ø	2	4-Ø-5-Ø-Ø-Ø-Ø
**A52**	4-3	IIaA15G2R1	4	14	5	8	18	37	16	7	4-14-5-8-18-37-16
**A53**	4-3	IIaA15G2R1	4	14	5	8	18	37	16	7	4-14-5-8-18-37-16
**A54**	4-3	IIaA15G2R1	4	14	5	8	18	37	16	7	4-14-5-8-18-37-16
**A55**	4-3	IIaA15G2R1	4	14	5	8	18	37	16	7	4-14-5-8-18-37-16
**A56**	4-3	IIaA15G2R1	4	14	5	8	18	37	16	7	4-14-5-8-18-37-16
**A57**	4-3	IIaA15G2R1	4	14	5	8	18	37	16	7	4-14-5-8-18-37-16
**A58**	4-3	IIaA15G2R1	4	14	5	8	18	37	16	7	4-14-5-8-18-37-16
**A59**	4-3	IIaA15G2R1	4	14	5	8	18	37	16	7	4-14-5-8-18-37-16
**A63**	5-1	IIaA15G2R1	4	14	5	8	18	37	16	7	4-14-5-8-18-37-16
**A65**	5-1	IIaA15G2R1	4	14	5	8	18	37	16	7	4-14-5-8-18-37-16
**A68**	5-2	IIaA15G2R1	4	Ø	5	Ø	Ø	Ø	16	3	4-Ø-5-Ø-Ø-Ø-16
**A76**	6-1	IIaA15G2R1	Ø	Ø	Ø	Ø	Ø	Ø	Ø	0	Ø-Ø-Ø-Ø-Ø-Ø-Ø
**A79**	6-1	IIaA15G2R1	Ø	Ø	5	Ø	Ø	Ø	Ø	1	Ø-Ø-5-Ø-Ø-Ø-Ø
**A102**	7-2	IIaA15G2R1	Ø	Ø	Ø	Ø	18	37	16	3	Ø-Ø-Ø-Ø-18-37-16

Supplementary Table S2B. MLVA profiles of post-partum cattle in Pen B.

Sample ID	Sample Point	Gp60 genotype	Loci / Number of repeats							Number of Loci	Final genotype
			cgd1	cgd4	MSF	cgd5	cgd6	cgd8	MM19		
**B21**	2-3	IIaA15R1	14	4	5	8	18	Ø	14	6	4-14-5-8-18-Ø-14
**B45**	3-3	IIaA14G2R1	Ø	Ø	Ø	Ø	Ø	Ø	Ø	0	Ø-Ø-Ø-Ø-Ø-Ø-Ø
**B46**	4-1	IIaA15G2R1	Ø	Ø	Ø	Ø	Ø	Ø	Ø	0	Ø-Ø-Ø-Ø-Ø-Ø-Ø
**B47**	4-1	IIaA15G2R1	4	14	5	8	18	37	16	7	4-14-5-8-18-37-16
**B48**	4-1	IIaA15G2R1	Ø	Ø	5	Ø	18	Ø	Ø	2	Ø-14-5-Ø-18-Ø-Ø
**B49**	4-1	IIaA15G2R1	4	Ø	Ø	Ø	18	37	Ø	3	4-Ø-Ø-Ø-18-37-Ø
**B50**	4-1	IIaA15G2R1	4	14	Ø	Ø	Ø	Ø	Ø	2	4-14-Ø-Ø-Ø-Ø-Ø
**B51**	4-1	IIaA15G2R1	Ø	Ø	Ø	Ø	Ø	Ø	16	1	Ø-Ø-Ø-Ø-Ø-Ø-16
**B52**	4-2	IIaA15G2R1	Ø	Ø	Ø	Ø	Ø	Ø	Ø	0	Ø-Ø-Ø-Ø-Ø-Ø-Ø
**B64**	5-1	IIaA15G2R1	4	14	5	Ø	Ø	37	Ø	4	4-14-5-Ø-Ø-37-Ø
**B68**	5-3	IIaA15G2R1	Ø	14	5	Ø	Ø	37	Ø	3	Ø-14-5-Ø-Ø-37-Ø
**B69**	5-3	IIaA15G2R1	Ø	Ø	5	Ø	Ø	Ø	Ø	1	Ø-Ø-5-Ø-Ø-Ø-Ø
**B87**	6-2	IIaA15G2R1	4	Ø	Ø	Ø	Ø	Ø	16	2	4-Ø-Ø-Ø-Ø-Ø-16
**B91**	6-2	IIaA15G2R1	4	14	5	8	18	37	16	7	4-14-5-8-18-37-16
**B97**	7-1	IIaA15G2R1	4	Ø	5	Ø	Ø	Ø	Ø	2	4-Ø-5-Ø-Ø-Ø-Ø
**B104**	7-2	IIaA15G2R1	Ø	Ø	Ø	Ø	Ø	Ø	Ø	0	Ø-Ø-Ø-Ø-Ø-Ø-Ø
**B105**	7-2	IIaA15G2R1	Ø	Ø	Ø	Ø	Ø	Ø	Ø	0	Ø-Ø-Ø-Ø-Ø-Ø-Ø
**B110**	7-2	IIaA15G2R1	Ø	Ø	Ø	Ø	Ø	37	16	2	Ø-Ø-Ø-Ø-Ø-37-16

Supplementary Table S2C. MLVA profiles of calves in Pen CA.

Sample ID	Sample Point	Gp60 genotype	Loci / Number of repeats							Number of Loci	Final genotype
			cgd1	cgd4	MSF	cgd5	cgd6	cgd8	MM19		
**CA14**	4-1	IIaA15G2R1	4	14	5	Ø	18	Ø	Ø	4	4-14-5-Ø-18-Ø-Ø
**CA15**	4-2	IIaA15G2R1	Ø	Ø	Ø	Ø	Ø	Ø	Ø	0	Ø-Ø-Ø-Ø-Ø-Ø-Ø
**CA16**	4-2	IIaA15G2R1	Ø	Ø	5	Ø	18	37	Ø	3	Ø-Ø-5-Ø-18-37-Ø
**CA19**	4-3	IIaA15G2R1	4	14	5	8	18	37	16	7	4-14-5-8-18-37-16
**CA20**	4-3	IIaA15G2R1	Ø	Ø	5	Ø	Ø	Ø	Ø	1	Ø-Ø-5-Ø-Ø-Ø-Ø
**CA21**	4-3	IIaA15G2R1	Ø	Ø	Ø	8	18	37	Ø	3	Ø-Ø-Ø-8-18-37-Ø
**CA24**	5-1	IIaA15G2R1	Ø	Ø	Ø	Ø	Ø	Ø	Ø	0	Ø-Ø-Ø-Ø-Ø-Ø-Ø
**CA25**	5-2	IIaA15G2R1	4	Ø	Ø	Ø	Ø	Ø	Ø	1	4-Ø-Ø-Ø-Ø-Ø-Ø
**CA27**	5-2	IIaA15G2R1	Ø	Ø	Ø	Ø	Ø	Ø	Ø	0	Ø-Ø-Ø-Ø-Ø-Ø-Ø
**CA34**	7-1	IIaA15G2R1	Ø	Ø	5	Ø	Ø	Ø	Ø	1	Ø-Ø-5-Ø-Ø-Ø-Ø
**CA35**	7-1	IIaA15G2R1	Ø	Ø	Ø	Ø	18	Ø	Ø	1	Ø-Ø-Ø-Ø-18-Ø-Ø
**CA36**	7-1	IIaA15G2R1	Ø	14	5	8	18	Ø	Ø	4	Ø-14-5-8-18-Ø-Ø
**CA37**	7-1	IIaA15G2R1	4	14	5	8	18	37	16	7	4-14-5-8-18-37-16
**CA38**	7-1	IIaA15G2R1	Ø	Ø	5	Ø	18	Ø	Ø	2	Ø-Ø-5-Ø-18-Ø-Ø
**CA39**	7-3	IIaA15G2R1	4	Ø	5	Ø	18	37	Ø	4	4-Ø-5-Ø-18-37-Ø
**CA40**	7-3	IIaA15G2R1	Ø	Ø	Ø	Ø	Ø	Ø	Ø	0	Ø-Ø-Ø-Ø-Ø-Ø-Ø
**CA41**	7-3	IIaA15G2R1	Ø	Ø	Ø	Ø	Ø	Ø	Ø	0	Ø-Ø-Ø-Ø-Ø-Ø-Ø
**CA42**	8-3	IIaA15G2R1	Ø	Ø	Ø	Ø	Ø	Ø	Ø	0	Ø-Ø-Ø-Ø-Ø-Ø-Ø
**CA44**	8-3	IIaA15G2R1	Ø	Ø	Ø	Ø	Ø	Ø	Ø	0	Ø-Ø-Ø-Ø-Ø-Ø-Ø
**CA45**	8-3	IIaA15G2R1	4	14	5	8	18	37	16	7	4-14-5-8-18-37-16
**CA46**	8-3	IIaA15G2R1	Ø	14	5	8	18	37	16	6	Ø-14-5-8-18-37-16

Sample ID	Sample Point	Gp60 genotype	Loci / Number of repeats							Number of Loci	Final genotype
			cgd1	cgd4	MSF	cgd5	cgd6	cgd8	MM19		
**CA47**	9-1	-	Ø	Ø	Ø	Ø	Ø	Ø	16	1	Ø-Ø-Ø-Ø-Ø-Ø-16
**CA48**	9-1	-	Ø	Ø	Ø	Ø	Ø	Ø	16	1	Ø-Ø-Ø-Ø-Ø-Ø-16
**CA49**	9-1	-	4	14	Ø	8	Ø	37	16	5	4-14-Ø-8-Ø-37-16
**CA50**	9-2	-	Ø	Ø	Ø	Ø	Ø	Ø	16	1	Ø-Ø-Ø-Ø-Ø-Ø-16
**CA56**	10-3	-	Ø	Ø	5	Ø	Ø	Ø	Ø	1	Ø-Ø-5-Ø-Ø-Ø-Ø
**CA59**	10-3	-	4	14	Ø	8	Ø	37	Ø	4	4-14-Ø-8-Ø-37-Ø
**CA60**	11-1	-	4	14	Ø	8	Ø	37	Ø	4	4-14-Ø-8-Ø-37-Ø
**CA61**	11-1	-	4	14	5	8	18	37	Ø	6	4-14-5-8-18-37-Ø
**CA91**	14-2	-	4	14	5	8	18	37	16	7	4-14-5-8-18-37-16
**CA47**	9-1	-	Ø	Ø	Ø	Ø	Ø	Ø	16	1	Ø-Ø-Ø-Ø-Ø-Ø-16

Supplementary Table S2D. MLVA profiles of calves in Pen CB.

Sample ID	Sample Point	Gp60 genotype	Loci / Number of repeats							Number of Loci	Final genotype
			cgd1	cgd4	MSF	cgd5	cgd6	cgd8	MM19		
**CB1**	10-2	IIaA15G2R1	4	14	5	8	18	37	16	7	4-14-5-8-18-37-16
**CB2**	10-2	IIaA15G2R1	4	14	5	8	18	37	16	7	4-14-5-8-18-37-16
**CB3**	10-2	IIaA15G2R1	4	14	5	8	18	Ø	16	6	4-14-5-8-18-Ø-16
**CB4**	10-2	IIaA15G2R1	4	Ø	5	8	18	Ø	16	5	4-Ø-5-8-18-Ø-16
**CB5**	10-2	IIaA15G2R1	4	14	5	8	18	37	16	7	4-14-5-8-18-37-16
**CB6**	10-3	IIaA15G2R1	4	14	5	8	18	37	16	7	4-14-5-8-18-37-16
**CB7**	10-3	IIaA15G2R1	4	14	5	8	18	37	16	7	4-14-5-8-18-37-16
**CB8**	10-3	IIaA15G2R1	4	14	5	8	18	37	16	7	4-14-5-8-18-37-16
**CB9**	10-3	IIaA15G2R1	4	14	5	8	18	37	16	7	4-14-5-8-18-37-16
**CB10**	10-3	IIaA15G2R1	4	14	5	8	18	37	16	7	4-14-5-8-18-37-16
**CB11**	11-1	IIaA15G2R1	4	Ø	Ø	8	Ø	Ø	16	3	4-Ø-Ø-8-Ø-37-16
**CB12**	11-1	IIaA15G2R1	4	14	Ø	8	18	37	Ø	5	4-14-Ø-8-18-37-Ø
**CB13**	11-1	IIaA15G2R1	4	14	5	8	18	37	16	7	4-14-5-8-18-37-16
**CB14**	11-1	IIaA15G2R1	4	14	5	8	18	37	16	7	4-14-5-8-18-37-16
**CB15**	11-1	IIaA15G2R1	4	14	5	8	18	37	16	7	4-14-5-8-18-37-16
**CB16**	11-2	IIaA15G2R1	4	14	5	8	18	37	16	7	4-14-5-8-18-37-16

Supplementary Table S2E. MLVA profiles of calves in Pen CC.

Sample ID	Sample Point	Gp60 genotype	Loci / Number of repeats							Number of Loci	Final genotype
			cgd1	cgd4	MSF	cgd5	cgd6	cgd8	MM19		
**CC1**	12-1	IIaA15G2R1	4	14	5	8	18	37	16	7	4-14-5-8-18-37-16
**CC2**	12-2	IIaA15G2R1	4	14	5	8	18	Ø	16	6	4-14-5-8-18-Ø-16
**CC4**	12-3	IIaA15G2R1	4	14	5	8	18	37	16	7	4-14-5-8-18-37-16
**CC5**	14-1	IIaA15G2R1	Ø	Ø	5	Ø	Ø	Ø	Ø	1	Ø-Ø-5-Ø-Ø-Ø-Ø
**CC7**	14-2	IIaA15G2R1	Ø	Ø	5	Ø	Ø	37	Ø	2	Ø-Ø-5-Ø-Ø-37-Ø
**CC8**	14-3	IIaA15G2R1	4	Ø	5	Ø	18	37	16	5	4-14-5-Ø-18-37-16
**CC9**	14-3	IIaA15G2R1	4	Ø	5	8	Ø	37	16	5	4-14-5-8-Ø-37-16
**CC10**	14-3	IIaA15G2R1	4	14	5	8	18	37	16	7	4-14-5-8-18-37-16
**CC11**	15-1	IIaA15G2R1	4	14	5	8	18	37	16	7	4-14-5-8-18-37-16
**CC12**	15-1	IIaA15G2R1	4	14	5	8	18	37	16	7	4-14-5-8-18-37-16

Supplementary Table S2F. MLVA profiles of calves in Pen CD.

Sample ID	Sample Point	Gp60 genotype	Loci / Number of repeats							Number of Loci	Final genotype
			cgd1	cgd4	MSF	cgd5	cgd6	cgd8	MM19		
**CD2**	15-1	IIaA15G2R1	4	Ø	5	8	18	Ø	16	5	4-Ø-5-8-18-Ø-16
**CD4**	15-3	IIaA15G2R1	Ø	Ø	Ø	8	Ø	Ø	Ø	1	Ø-Ø-Ø-8-Ø-Ø-Ø
**CD5**	15-3	IIaA15G2R1	Ø	Ø	5	8	18	37	16	5	Ø-Ø-5-8-18-37-16
**CD6**	15-3	IIaA15G2R1	Ø	Ø	Ø	Ø	Ø	Ø	Ø	0	Ø-Ø-Ø-Ø-Ø-Ø-Ø
**CD8**	15-3	IIaA15G2R1	Ø	Ø	Ø	Ø	Ø	Ø	Ø	0	Ø-Ø-Ø-Ø-Ø-Ø-Ø
**CD9**	16-1	IIaA15G2R1	4	Ø	Ø	Ø	Ø	Ø	16	2	4-Ø-Ø-Ø-Ø-Ø-16
**CD10**	16-1	IIaA15G2R1	4	14	5	8	18	37	16	7	4-14-5-8-18-37-16

Supplementary Table S2G. MLVA profiles of calves in Pen CE.

Sample ID	Sample Point	Gp60 genotype	Loci / Number of repeats							Number of Loci	Final genotype
			cgd1	cgd4	MSF	cgd5	cgd6	cgd8	MM19		
**CE2**	15-3	IIaA15G2R1	4	Ø	Ø	Ø	Ø	Ø	16	2	4-14-Ø-Ø-Ø-Ø-16
**CE3**	16-1	IIaA15G2R1	4	Ø	5	8	18	Ø	Ø	4	4-Ø-5-8-18-Ø-Ø
**CE4**	16-2	IIaA15G2R1	4	14	5	8	18	37	16	7	4-14-5-8-18-37-16
**CE8**	16-3	IIaA15G2R1	4	14	5	8	18	37	16	7	4-14-5-8-18-37-16
**CE12**	17-1	IIaA15G2R1	4	14	5	8	18	37	16	7	4-14-5-8-18-37-16
**CE13**	17-1	IIaA15G2R1	Ø	Ø	5	Ø	18	Ø	Ø	2	Ø-Ø-5-Ø-18-Ø-Ø
**CE14**	17-1	IIaA15G2R1	4	14	5	8	18	37	16	7	4-14-5-8-18-37-16

Supplementary Table S2H. MLVA profiles of calves in Pen D.

Sample ID	Sample Point	Gp60 genotype	Loci / Number of repeats							Number of Loci	Final genotype
			cgd1	cgd4	MSF	cgd5	cgd6	cgd8	MM19		
**D11**	3-2	-	Ø	Ø	Ø	Ø	Ø	Ø	Ø	0	Ø-Ø-Ø-Ø-Ø-Ø-Ø
**D13**	3-2	-	Ø	Ø	Ø	Ø	Ø	Ø	Ø	0	Ø-Ø-Ø-Ø-Ø-Ø-Ø
**D14**	3-2	-	Ø	Ø	Ø	Ø	Ø	Ø	Ø	0	Ø-Ø-Ø-Ø-Ø-Ø-Ø
**D15**	3-2	-	Ø	Ø	Ø	Ø	Ø	Ø	Ø	0	Ø-Ø-Ø-Ø-Ø-Ø-Ø
**D19**	4-1	IIaA15G2R1	Ø	Ø	5	Ø	18	Ø	Ø	2	Ø-Ø-5-Ø-18-Ø-Ø
**D20**	4-1	IIaA15G2R1	4	14	5	8	18	37	16	7	4-14-5-8-18-37-16

Supplementary Table S2I. MLVA profiles of calves in Pen E.

Sample ID	Sample Point	Gp60 genotype	Loci / Number of repeats							Number of Loci	Final genotype
			cgd1	cgd4	MSF	cgd5	cgd6	cgd8	MM19		
**E1**	5-2	IIaA15G2R1	Ø	Ø	5	Ø	Ø	Ø	Ø	1	Ø-Ø-5-Ø-Ø-Ø-Ø
**E3**	5-2	IIaA15G2R1	4	Ø	5	Ø	18	37	16	5	4-Ø-5-Ø-18-37-16
**E4**	5-2	IIaA15G2R1	Ø	Ø	5	Ø	Ø	Ø	Ø	1	Ø-Ø-5-Ø-Ø-Ø-Ø
**E6**	5-3	IIaA15G2R1	4	14	5	8	18	37	16	7	4-14-5-8-18-37-16

Supplementary Table S2J. MLVA profiles of calves in Pen G.

Sample ID	Sample Point	Gp60 genotype	Loci / Number of repeats							Number of Loci	Final genotype
			cgd1	cgd4	MSF	cgd5	cgd6	cgd8	MM19		
**G1**	11-3	IIaA15G2R1	Ø	Ø	5	Ø	Ø	Ø	Ø	1	Ø-Ø-5-Ø-Ø-Ø-Ø
**G2**	11-3	IIaA15G2R1	Ø	Ø	Ø	Ø	Ø	Ø	Ø	0	Ø-Ø-Ø-Ø-Ø-Ø-Ø
**G3**	12-1	IIaA15G2R1	4	14	5	8	18	37	16	7	4-14-5-8-18-37-16
**G4**	12-1	IIaA15G2R1	4	14	5	8	18	37	16	7	4-14-5-8-18-37-16
**G5**	12-1	IIaA15G2R1	Ø	Ø	5	8	18	37	16	5	4-Ø-5-8-18-37-16
